# First Evaluation of the TREPP Procedure for Inguinoscrotal Hernia Repair: A Step-Up Study

**DOI:** 10.3389/jaws.2025.15098

**Published:** 2025-09-03

**Authors:** T. L. R. Zwols, M. J. W. Möllers, N. J. G. M. Veeger, H. H. Eker, G. G. Koning, J. P. E. N. Pierie

**Affiliations:** ^1^ Department of Surgery, Isala Zwolle, Zwolle, Netherlands; ^2^ Postgraduate School of Medicine, University Medical Center Groningen, Groningen, Netherlands; ^3^ Department of Surgery, Nij Smellinghe Hospital, Drachten, Netherlands; ^4^ MCL Academy, Frisius MC, Leeuwarden, Netherlands; ^5^ Department of Epidemiology, University Medical Center Groningen, University of Groningen, Groningen, Netherlands; ^6^ Department of Surgery, University Hospital, Ghent, Belgium; ^7^ Department of Surgery, Euregio Hospital, Nordhorn, Germany; ^8^ Department of Surgery, Frisius MC, Leeuwarden, Netherlands

**Keywords:** TREPP, inguinoscrotal, preperitoneal, hernia, scrotal

## Abstract

**Background:**

Inguinoscrotal hernias, classified as large indirect hernias (L3) within the European Hernia Society classification, pose unique surgical challenges. The TransREctus sheath Pre-Peritoneal (TREPP) repair is an extraperitoneal approach that may offer advantages over conventional techniques, such as reduced intra-abdominal complications and the potential to avoid general anesthesia. However, its use in inguinoscrotal hernia repair was to date not explored.

**Methods:**

This retrospective study analyzed consecutive patients who underwent TREPP repair for scrotal hernia between January 2021 and December 2023. Patient data were extracted from an electronic hospital database, and follow-up was conducted via patient records and the PINQ-PHONE questionnaire. Primary outcomes included recurrence rates, postoperative pain, and surgical complications.

**Results:**

A total of 33 primary scrotal hernia cases were analyzed. The majority of procedures (87.9%) were performed under spinal anesthesia, with a median operative time of 23 min for unilateral cases. No recurrences were observed within the follow-up period of at least 1 year. One patient (3.0%) reported persistent postoperative pain, successfully managed with local anesthetic and corticosteroid injections. Two patients (6.1%) developed infections, treated conservatively with antibiotics. Other minor complications included postoperative hematoma (9.1%), seroma (9.1%) or urinary retention (6.1%), all managed without surgical intervention.

**Conclusion:**

The TREPP procedure in experienced hands appears to be a feasible and safe alternative for scrotal hernia repair, demonstrating low complication rates requiring reintervention and no recurrences in this cohort. Despite the study’s retrospective design and small sample size, this first results step-up study support further investigation into the role of TREPP repair in inguinoscrotal hernia management. Larger, prospective studies are needed to confirm its long-term efficacy.

## Introduction

Inguinal hernias in which abdominal contents, such as the intestine, descend into the scrotum are called scrotal hernias. Within the European Hernia Society groin hernia classification, they are a subdivision of large indirect hernias (L3) [1]. The size of these scrotal hernias can vary widely, and a recent suggestion from the HerniaSurge collaborative is to categorize in S1 (upper third of thigh), S2 (middle third of thigh) and S3 (lower third of thigh/patellar), measuring to the lowest part of the scrotum in a standing position [2]. Another, recently published classification follows a similar division in zones (Z1 to Z5), also classifying hernias within the groin area and hernias below the knee [3]. Scrotal hernias can result in significant discomfort, pain and complications such as strangulation, requiring direct surgical intervention.

The standard treatment for symptomatic scrotal hernias is surgical repair [4]. Over the years, several techniques have been developed to achieve durable hernia repair while minimizing complications and recurrence. Traditional open approaches, such as Lichtenstein repair, and endoscopic methods, like transabdominal preperitoneal (TAPP) and totally extraperitoneal (TEP) repairs, have been widely used for inguinal hernia repair and, by extension, in scrotal hernia repair [4]. In most cases, a Lichtenstein procedure is performed after perioperative reduction of the scrotal hernia. While these techniques are effective for most inguinal hernias, they can be more technically challenging in (large) scrotal hernias due to the extensive dissection required and are accompanied by an increased risk of postoperative complications [5].

Since 2006, a newer approach known as the TransREctus sheath Pre-Peritoneal (TREPP) mesh repair has emerged as a promising alternative for the management of inguinal hernias. This procedure involves an extraperitoneal approach, offering several advantages over traditional methods, including reduced risk of intra-abdominal complications and better biomechanical positioning of the mesh, while avoiding the necessity for general anesthesia [6–10]. Despite these promising benefits, the TREPP technique is not yet widely adopted, and to date the evidence regarding its outcome in all types of hernia still remains limited.

The aim of this study is to evaluate the clinical outcome of the TREPP procedure specifically in the treatment of scrotal hernias. By analyzing surgical results of our patients retrospectively, such as postoperative recovery, postoperative pain and recurrence rates, we aim to provide further insight into the feasibility and efficacy of TREPP repair for scrotal hernia. Understanding the role of TREPP in scrotal hernia management could contribute to optimizing surgical strategies and improving patient outcomes.

## Methods

We performed a search in the electronic patient database of our hospital, in which all consecutive operative records containing “scrotal” and “hernia” in the operation code or surgical conclusion were selected. All scrotal hernias in our population were categorized as S1 (upper third of thigh). The TREPP procedure was performed by one experienced TREPP surgeon following the 9 steps principle [6]. When the hernia sac could not be fully repositioned, it was ligated and transected, leaving the distal part *in situ*. Either a medium (8.6 cm x 14.2 cm) or a large (10.2 cm x 15.7 cm) OnFlex™ Mesh (BD, United States of America) was used, depending on the size of the preperitoneal space.

The timeframe of the search was from January 1st^,^ 2021 (start of implementation of TREPP for scrotal hernia repair) until December 31st^,^ 2023. All selected patient files were viewed for perioperative or postoperative complications, up until the date of review, which was 1 year after the last included surgery. All consecutive patients with primary scrotal hernia for which a TREPP procedure was performed were included in the analysis. Patients were called and were asked questions from the PINQ-PHONE questionnaire. This validated questionnaire assesses the probability of recurrence hernia [11]. An additional generic pain evaluation by means of the numeric rating scale (NRS) was performed. Outcome measurements were: diagnosed recurrence, postoperative pain (defined as any extra visits or treatments for pain in the patient record) and infectious complication (wound infection, abscess). If any complication required secondary intervention, this was reported as well. Data were analyzed in SPSS Statistics for Windows, version 22. This study was reported using the Strengthening the Reporting of Observational Studies in Epidemiology (STROBE) Statement: guidelines for reporting observational studies [12].

## Results

A search in the electronic patient records resulted in a total of 34 procedures in which a scrotal hernia was treated with the TREPP procedure. Of these, 33 procedures were performed for a primary scrotal hernia, one patient was treated because of recurrent scrotal hernia and was excluded from further analysis. Within these included 33 patients, one patient had a bilateral scrotal hernia and six patients had a bilateral hernia, of which only one side was scrotal. Patient characteristics are shown in Table 1.

**TABLE 1:** Patient and procedure characteristics.

	Prevalence (*n* = 33)	Range/percentage
Age^a^ (mean; in years)	68	range 18–90
ASA classification		
1 2 3 4	5 19 9 0	15.2% 57.6% 27.3% 0%
BMI (kg/m^2^)	27	range 21–41
Side of hernia		
Left	11	33.3%
Right	15	45.5%
Bilateral (one side scrotal)	6	18.2%
Bilateral (two sides scrotal)	1	3.0%
Duration of surgery (minutes)^b^	23	range 12–39
Duration of surgery in bilateral hernia (minutes)	44	range 37–68
Anesthesia type		
Spinal	29	87.9%
General	4	12.1%

^a^ At time of operation.

^b^ Calculation based upon single sided procedures (*n* = 25). In one procedure there was no operation time recorded in the electronic record.

After analyzing patient records for outcome of surgery, 22 out of 33 patients (66.7%) were free from any complication up until analysis (range 1–3 years postoperatively). There were no recurrences within the study population within our postoperative time window, varying from one to 3 years after surgery. One patient (3.0%) developed postoperative pain, interpreted as a neuropathy of the 12th thoracic nerve due to anterior cutaneous nerve entrapment syndrome (ACNES) in the scar tissue. This patient required two consecutive injections of a local analgesic (bupivacaine) and a local corticosteroid (triamcinolone acetonide), following treatment principles as described in the ACNES guideline, after which pain complaints resolved completely. Two patients (6.1%) developed a superficial infection, which was treated conservatively with antibiotics. There was no prevalence of mesh infections. All adverse events are shown in Table 2.

**TABLE 2:** Overview of procedure related complications.

Adverse events	Prevalence*n* = 33	%
None	22	66.7
Recurrence	0	0
Postoperative pain	1	3.0
Local adverse events		
Infectious	2	6.1
Bleeding^a^	3	9.1
Seroma	3	9.1
Urinary retention	2	6.1

^a^ Both “haematoma” and “postoperative bleeding” were used in electronic patient records. No bleeding complications led to re-surgery.

After analysis of the electronic patient records patients were called and asked to participate in the PINQ-PHONE questionnaire. Two patients were deceased (no relation to the surgery) and 21 patients (67.7% response rate) were reached after multiple telephone attempts. 15 out of 21 patients (71.4%) responded with “no” on all questions, reported a NRS of 0, and expressed satisfaction with the result. One of the patients (4.7%) responded with “yes” on question one, two and three, as well as a NRS of 7. Five other patients reported feeling “something” in their operated groin, but felt no swelling and could not describe this as pain or discomfort.

## Discussion

The treatment of scrotal hernias remains a challenging surgical endeavor. Current guidelines do not provide one decisive answer as to the most optimal surgical technique for scrotal hernia repair. Traditional techniques, while effective, often carry a risk of complications or recurrence, particularly when dealing with the complex anatomy of a scrotal hernia.

Our study, although limited by a small sample size and its retrospective design, provides the first promising results of using the TransREctus sheath Pre-Peritoneal (TREPP) procedure in the treatment of scrotal hernias.

Although scrotal hernia surgery in general is associated with a higher prevalence of complications [5], these early evaluations suggest that the TREPP repair results in comparable rates of postoperative complications. A recent study evaluating outcome in scrotal hernia repair with a different open preperitoneal technique also shows a significantly higher percentage of any complication compared to non-scrotal hernias [13]. Comparable to these previous studies, our population with scrotal hernias shows a relatively high age, BMI and ASA classification. The lack of major adverse events and the absence of recurrence observed in this cohort support the potential safety and efficacy of this technique, even in cases of more complex scrotal hernias.

However, there are several limitations to consider when interpreting these findings. First, the small number of patients included in the study limits the generalizability of our results. Scrotal hernias are less common than other forms of inguinal hernia, and thus larger (multicenter) studies will be required to validate the outcomes observed here. Second, the retrospective nature of the study introduces potential biases, such as incomplete data capture or selection bias, which may have influenced the results. Lastly, the response rate for the PINQ-PHONE questionnaire by telephone was relatively low, allowing for potential underreporting of long-term complications such as recurrence or pain.

Despite these limitations, our findings demonstrate feasibility of the TREPP procedure for scrotal hernias. Adhering to the IDEAL (Idea, Development, Evaluation, Assessment, Long-term study) recommendations, this study can be categorized in the development/exploration phase, in which different indications are investigated [14]. As this technique continues to evolve, further research is necessary to refine patient selection criteria and optimize surgical outcomes. Prospective studies with larger cohorts and longer follow-up periods are particularly needed to confirm the safety and effectiveness of the TREPP procedure for scrotal hernia repair in comparison to other established surgical methods.

In conclusion, while the level of evidence of our study is limited (Level of Evidence 4) [15], this step-up study shows that the TREPP procedure can be performed for inguinoscrotal hernia repair. It appears to be a safe and feasible option for the treatment of scrotal hernias, and most likely can be added to the inguinal hernia surgeon’s armament after sufficient training. Future research should aim to clarify the long-term benefits and potential risks associated with this technique in order to guide clinical practice and improve patient outcomes.

## Data Availability

The raw data supporting the conclusions of this article will be made available by the authors, without undue reservation.
